# Recent Trends in Magnetic Polymer Nanocomposites for Aerospace Applications: A Review

**DOI:** 10.3390/polym14194084

**Published:** 2022-09-29

**Authors:** David Romero-Fierro, Moises Bustamante-Torres, Francisco Bravo-Plascencia, Aylin Esquivel-Lozano, Juan-Carlos Ruiz, Emilio Bucio

**Affiliations:** 1 Departamento de Química de Radiaciones y Radioquímica, Instituto de Ciencias Nucleares, Universidad Nacional Autónoma de México, Circuito Exterior, Ciudad Universitaria, Ciudad de Mexico 04510, Mexico; 2 Departamento de Biología, Escuela de Ciencias Biológicas e Ingeniería, Universidad de Investigación de Tecnología Experimental Yachay, Hacienda San José s/n y Proyecto Yachay (Ciudad del Conocimiento Yachay), Urcuquí 100650, Ecuador; 3 Centro Conjunto de Investigación en Química Sustentable UAEM-UNAM, Toluca 50200, Mexico; 4 Instituto de Química, Universidad Nacional Autónoma de México, Circuito Exterior, Ciudad Universitaria, Coyoacán, Ciudad de Mexico 04510, Mexico; 5 Process and Technology Department, Division of Natural Science and Engineering, Universidad Autonoma Metropolitana-Cuajimalpa, Ciudad de Mexico 05300, Mexico

**Keywords:** polymer nanocomposite, filler, magnetic nanoparticles, aerospace devices, nanotechnology

## Abstract

Polymers have had an enormous impact on science and technology, and their interest relating to the development of new macromolecular materials has exponentially increased. Polymer nanocomposites, materials based on a polymeric matrix covalently coupled to reinforcement, display properties of both components. In the aerospace industry, polymer nanocomposites are attractive due to their promising characteristics, among which lightness, mechanical and thermal resistance, radiation and corrosion resistance, and conductive and magnetic properties stand out. The use of them, instead of metal-based materials, has allowed the optimization of design processes and applications in order to provide safer, faster, and eventually cheaper transportation in the future. This comparative review collects the most relevant and prominent advances in the development of polymer nanocomposites with aerospace applications starting from basic aspects such as the definition of polymer nanocomposite to more specialized details such as synthesis, characterization, and applications, in addition to proposing new research branches related to this topic.

## 1. Introduction

The aerospace industry requires components made of materials capable of meeting requirements such as lightness, excellent thermal resistance, high durability, and capable of resisting electromagnetic interference (EMI). Few materials manage to meet all these characteristics. Previously, metals were used to manufacture parts for this industry, although they were not the perfect candidates since they did not meet all the requirements such as lightness and low corrosion resistance. New materials have been designed that are capable of satisfying these needs, either by modifying already established materials or by combining two or more materials into one, called composites [1].

One of the most widely used materials for this purpose is polymers. This material has certain advantages over others due to its low costs, various synthesis, and the wide range of properties that it can provide, such as lightness, electrical conduction, high thermal resistance, wear, and corrosion, among others [2]. When the composites are made up of a polymeric matrix and a reinforcing material, which can be in the form of nanometric-sized fibers, particles, or flakes [3], and provides magnetic properties, then it is called a magnetic polymeric nanocomposite. Nanotechnology involves materials science and engineering, transforming many sectors, such as aerospace, energy, information technology, electronics, textiles, waste management, medicine, and transportation. This discipline will allow the development of new, more robust, lighter, and more durable materials with better chemical, physical, and thermal properties compared to the materials currently used [4,5].

Generally, metallic nanoparticles (MNPs) are used to reinforce the nanocomposite. Nanoparticles (NPs) are microscopic particles with a dimension of less than 100 nm [6], studied in the nanotechnology field. These MNPs are an exciting class of metal oxides that an external field can magnetize; they also have some electrical conductivity, closely related to EMI resistance’s effectiveness [7]. That is why using polymeric nanocomposites reinforced with MNPs represents a great advantage for aerospace applications. Besides, MNPs can be functionalized or encapsulated by organic or inorganic compounds [5,8], forming a nanocomposite.

There are many ways to synthesize nanocomposite materials. They will vary according to the location where the composite material is used, the shape and size of the composite material, the type of reinforcement material, and the availability of resources, such as the grafting method, blending, chemical vapor deposition (CVD), in situ precipitation, and molding. Furthermore, significant advances have been made in the design and handling of nanoparticles in a polymeric matrix, which allows more effective and selective synthesis [9]. Likewise, it is necessary to carry out an extensive study on improved properties that the new material possesses, through various characterization techniques such as Atomic Force Microscopy (AFM) and Transmission Electronics Microscopy (TEM) that help to determine the size of the reinforcing nanomaterial. Another important aspect to study is the impact that the reinforcement has on the matrix in terms of mechanical properties, since the incorporation of a new material influences the hardness, resistance to wear, and traction, observing that this confers an improvement on the composite compared to material without reinforcement [10]. 

**Figure 1:**
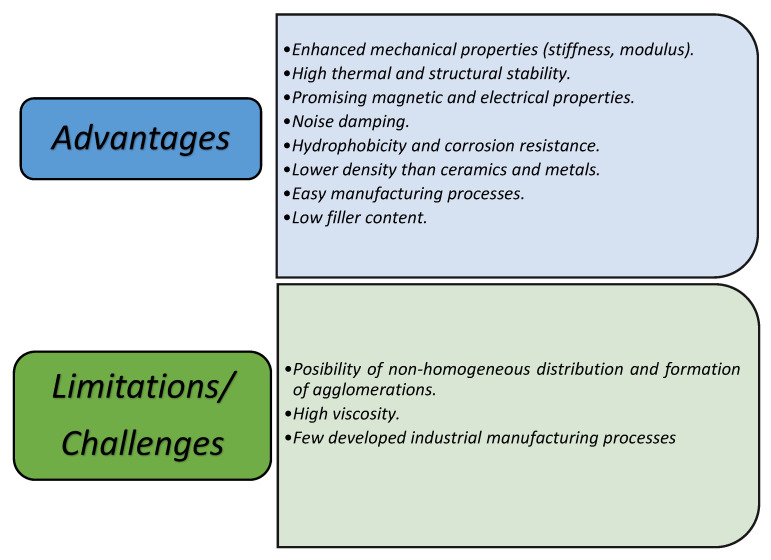
Advantages and limitations of polymer nanocomposites.

In order to provide a compendium of updated and innovative information for the study of magnetic polymeric composites, this paper presents the most promising results in this area. This is reflected in the different devices applicable to different areas, mainly in the aerospace industry, and the most used methods in the synthesis of these materials, as well as the essential characterization techniques. We take into account all the advantages offered by polymers over other materials and the implementation of nanotechnology, due to the very varied properties and applications of MNPs in different areas. This synergic combination provides better mechanical, thermal, barrier, and electromagnetic properties. These novel properties are well suited to the aerospace industry, making great contributions to protection against electromagnetic interference (EMI), corrosion, and structural health monitoring (SHM). Finally, taking into account all of these aspects, the following Figure 1 shows the advantages and disadvantages (challenges) of the use of polymer matrix nanocomposites in the aerospace industry.

## 2. Magnetic Polymer Nanocomposites (MPNs)

Polymers, metals, and ceramics are used as matrix materials for composite materials [11]. In short, the matrix is the structure that holds the reinforcement material. The most common and commercially available matrix materials used to make polymer composites are polyester resin, epoxy resin, and vinyl ester resin [12]. Reinforcement materials transmit the loads to the matrix; therefore, they define most of the mechanical characteristics of the material, such as resistance and rigidity, as well as magnetic, optical, and electrical properties of the final composite material, reducing costs [13]. Composite materials improve the aircraft system by reducing the aircraft’s weight, thereby decreasing the fuel cost per passenger. It is important to note that the three most common composite materials available are reinforced with glass fiber, carbon fiber, and aramid fiber. Currently, MNPs are frequently studied as part of a composite for airplane purposes [4].

Reinforcement can make up 20–80% of the volume of the composite material, but usually less than 50% [14]. In the simplest case, the appropriate addition of reinforcement to a polymeric matrix creates a functional material and it can improve its performance, often dramatically, simply by taking advantage of the nanoscale nature and properties of the filler [15,16,17]. Among the nanosized fillers, iron oxides have attracted significant interest, and magnetite (Fe_3_O_4_) and maghemite (γ-Fe_2_O_3_) especially have found numerous potential applications in magnetic recording technology, pigments, catalysis, photocatalysis, medical uses, and environmental processes [18].

MPNs materials constitute a new generation of multifunctional materials that combine the properties of conventional polymers and magnetic materials (ferrimagnetic and/or ferromagnetic particles mixed or embedded in a matrix) are classified as “magneto-polymeric materials” [19] or also known as ferrogels [20]. MPNs typically contain nanosized magnetic materials to trigger the response to an external stimulus (i.e., an external static or alternating magnetic field) [21]. Within ferrogels, elongation, contraction, and curvature can be induced by applying a homogeneous magnetic field [19].

Ferrites are ceramic-like compounds derived from iron oxides combined with metallic elements. These types of materials are non-conductive and ferrimagnetic. Due to their excellent magnetic and dielectric properties, ferrites are considered one of the best magnetic materials to be used with polymeric matrices in electromagnetic wave absorption applications [22]. This material’s carbon-based reinforcements are graphite, carbon fibers, carbon nanotubes, and graphene sheets. As a general trend, it is found that increasing the reinforcement content within a matrix increases the electrical conductivity, which is a fundamental factor for shielding and absorbing electromagnetic waves [23]. 

The inclusion of nanoparticles of inorganic origin dispersed in a polymeric matrix helps to improve mechanical, magnetic, optical, and even thermal properties [24]. However, this improvement will largely depend on the type of dispersion, which can be homogeneous or diffuse [25]. Li et al. modified the surface of poly(vinylidene fluoride-hexafluoropropylene) with BaTiO_3_ nanofibers (BT NFs). The results showed that the nanocomposite with 3 vol% BT NFs has an improved discharged energy density of 8.55 J/cm^3^ in an applied electric field (300 MV/m). This value is 43% higher than the pristine polymer matrix. This improvement is suitable for aerospace power systems, where, usually, high energy densities are achieved at ultrahigh applied electric fields (>400 MV/m) [26]. A recent study by Li et al. showed that the polarity of polymer shells on BaTiO_3_ nanoparticles has an interesting effect on the dielectric and energy storage of dielectric polymer nanocomposites [27].

MPNs can be synthesized by embedding magnetic particles into a non-magnetic (polymer) matrix, selected according to the technology used for fabricating the printed magnetic parts (e.g., polymer matrix, curable hydrogel, or solvent) [28]. To create exceptionally proficient attractive materials, the “doping” of polymer materials with attractive MNPs, made of an inorganic issue (frequently superparamagnetic press oxide Fe_3_O_4_ or γ-Fe_2_O_3_, or “delicate” metallic iron, yet additionally “hard” attractive materials, e.g., Ni, Co, FeN, FePd, FePt, etc.), gave off an impression of being a more engaging and effective arrangement [29]. These classes of material receive significant attention due to their potential in the fields of catalysis, sensor, enzyme immobilization, DNA extraction, drug delivery [30,31], bioremediation [32], and aerospace [23].

The massive development of polymers has driven their use in various fields such as aerospace, marine, automotive, military, and structural applications. The critical factor for using any composite material is performance and the degree of deterioration it may have. These factors will largely depend on the duration and interaction with your environment. The deterioration of polymeric materials will involve changes in their composition, both chemical and physical, due to the multiple reactions that can occur, including the cleavage of important bonds within the macromolecule. These changes lead to materials with very different characteristics, which are generally worse than the original material. These deteriorated materials will not contribute to the mechanical properties, and their useful life will be gradually diminished. For this reason, some polymers or its compound used outdoors must respond effectively to environmental conditions [33].

Thermal stability and flame retardancy are among the most important properties that a polymer or composite with aerospace applications must meet. Research in this field indicates that specifically modified epoxy nanocomposites have better flame properties than conventional composite materials [34]. According to the Kissinger method, the activation energies for the thermo-oxidative degradation process of modified materials are lower than pure epoxy in the first degradation stage. However, in the second thermo-oxidative stage, an inverse behavior is seen. In the same vein, it has been found that carbon nanotubes tend to outperform nanoclays as effective flame-retardant additives if the carbon nanoparticles form a network anchored to the polymer matrix so that the composite material behaves similar to a gel.

Thermal degradation of composite materials depends on the charge of the clay, the composition, and the nature of the ambient gas. A literature review by Leszczynska et al. analyzed the thermal stability of various matrices modified by montmorillonite clay and its influence factors [35]. Polymers have a hydrophobic character, so the clay must be modified with a surfactant that allows it to interact with the polymer [36]. Likewise, certain factors were found that influence the thermal stability of nanocomposites, such as the chemical constitution of the modifier, the chemical character of the polar compatibilizers, the natural thermal resistance of the matrix, the composition of the nanofiller, and also the access to oxygen of the material during the heating process. The clay is modified using ammonium salts as surface-active agents. Thus, the quaternary ammonium ion is commonly used to allow the adhesion of the silicate with a polymeric resin [37].

In general, including clay in a polymeric matrix considerably improves performance since it acts as a superior insulator and a mass transport barrier for the products generated during decomposition. In this sense, clay acts as a thermal barrier, which can be used to improve the thermal stability of the composite and help the formation of carbon during the decomposition process [3]. It is important to note that the temperature at which volatilization is generated in a nanocomposite is much higher than in its similar scal (microcomposite). Additionally, the thermal oxidation of the polymer undergoes a significant decrease in a nanocomposite that presents a high carbonization performance. This effect can be produced both by a catalytic action due to the presence of the silicate and the sites created by thermal decomposition, and by the barrier effect between both environments [37].

Studying a polymer’s performance and degradation is vital for the scientific community and the industry since its understanding will increase the composite’s useful life. Polymer degradation is caused by biodegradation, oxidation, mechanical and catalytic degradation, and pyrolysis. Due to their molecular structure, polymers are susceptible to harmful environmental changes. It should be emphasized that due attention has not been paid to the durability and performance of polymeric nanocomposites in terms of their synthesis processes and the evaluation of their mechanical properties [38].

Regarding the performance of polymeric nanocomposites, several studies have been carried out. The addition of clay to epoxy was found to decrease the curing reactivity of the resin. This involves a process that reduces the crosslinking density of the cured resin and the greater length of the polymer chains that lie between the crosslinking points [39]. Having longer polymer chains (which are less thermodynamically stable) together with smaller chains produces a composite with a greater tendency to degrade [40]. On the other hand, the silicate layers turn out to be barrier phases to common gases such as oxygen and nitrogen, thus allowing the material to isolate itself in the presence of these gases and extend its degradation time [41]. It was found that nanocomposites intercalated with 10 wt.% clay predominate the first mentioned factor, while in exfoliated materials (2 wt.% clay), the second is more important [23]. For this reason, it can be concluded that exfoliated materials have better barrier properties than intercalated ones [15].

**Figure 2:**
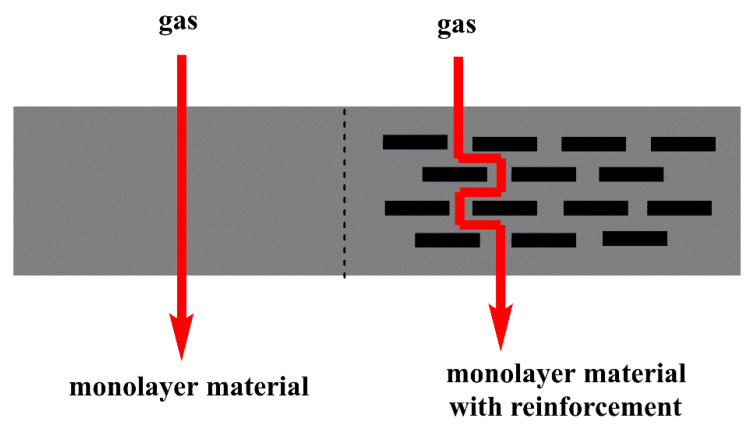
Gas diffusion through a matrix with and without nanoparticles.

The inclusion of nanoparticles can improve the barrier properties of material since the shape, size, degree of dispersion, and especially type of the particle–polymer interaction affect the transport properties of gases [42]. The inorganic particles added to a polymeric matrix constitute an impermeable phase; they represent a physical diffusion barrier, making the path that the gases must travel to cross the material longer, as shown in Figure 2. They can also act as nucleating agents, which affects the polymer crystals’ size and shape and restricts the movement of the chains. Models have been developed to explain and predict particle addition´s effect on permeability. In general, these are based on the effect of the particles on the tortuosity of the membrane since it is considered that the properties and characteristics of the polymeric matrix are not affected by the filling [43].

Regarding the magnetic properties of nanocomposites, it is essential to point out that organic–inorganic interactions add new properties that are impossible to obtain in organic or inorganic materials by themselves and provide a great capacity for transformation. The literature mainly shows two areas of magnetic nanocomposites’ application, as seen in the main nanotechnology initiatives in the United States and the European Community. One of them is the field of biomedicine, and the other one is the development of methods for manufacturing nano-organized structures and their applications and recording devices, sensors, and structures.

Polymeric nanocomposites are good candidates for applying of ferromagnetic nanoparticles on their surface as they have an extraordinary ability to be used as templates and in self-assembly. For example, the ordering can be achieved by self-assembling nanoparticles coated with a surfactant or in situ growth of the particles in a block copolymer as a template [44].

Functional materials are fundamental ingredients in the design of modern sensors and devices. Some of the many possibilities of combining magnetism with other properties in a polymer nanocomposite are:

- **Magnetic conductive materials:** These are useful in manufacturing sensors and devices. They are made up of magnetic nanoparticles in a conductive polymeric matrix. A charge transfer can be established between the surface of the particles and the polymer, so the material acts as an electronic system. Some proposed compositions are magnetite-polyaniline, maghemite-polypyrrole, cobalt ferrite-polypyrrole, and various metal-polymer combinations [45,46]
- **Transparent magnetic materials:** As magnetic oxides are considerably more transparent to visible light than nanoparticles, magnetic nanocomposites can be made with reasonable transparency and greater magnetization, by more than an order of magnitude, than stronger ones such as transparent magnets.

## 3. Synthesis of Magnetic Polymer Nanocomposites

The most efficient approach to induce internal order within polymer matrices by applying external magnetic fields is through the incorporation of MNPs [47]. Several processes have been designed to fabricate MNCs, such as molding [24], co-precipitation [48], in situ precipitation [49], blending [50], and the grafting method [51]. Here, we detail each process and some of the prominent examples.

### 3.1. Molding

**Figure 3:**
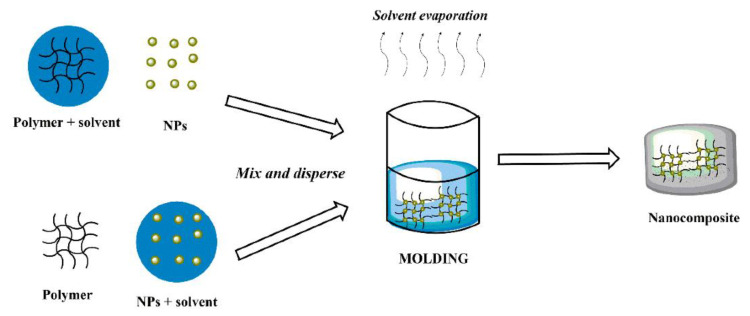
Schematic representation of molding method. Reprinted with permission from ref. [57], MDPI, 2022.

Molding is a conceptually simple form of transferring patterns by soft lithography [52]. It is a widely used fabrication process of MNCs that is accomplished by mixing magnetic fillers and polymeric precursors thoroughly and curing them to form specific shapes or structures in molds [24] (Figure 3). There are several types of molding processes, such as injection [53], resin transfer [54], and compression molding techniques [55], that practically use a mold that is completely filled under pressure and temperatures. The mold walls are heated to a temperature above the melting point of the mold material allowing a faster flow of material through the cavities [56].

### 3.2. Coprecipitation

**Figure 4:**
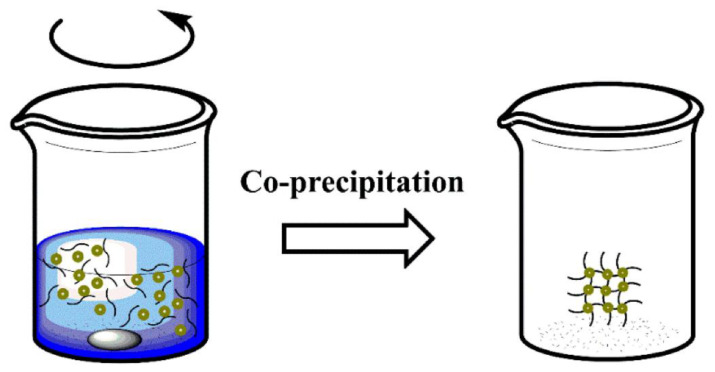
Schematic representation of co-precipitation method. Reprinted with permission from ref. [57], MDPI, 2022.

Coprecipitation is a very facile and convenient way to synthesize iron oxide nanoparticles (either Fe_3_O_4_ or γ-Fe_2_O_3_) from aqueous Fe^2+^/Fe^3+^ salt solutions by the addition of a base under an inert atmosphere at room temperature or at elevated temperature [58] (Figure 4). It is highly important to study and control the reaction parameters (e.g., type of metal cation precursors, molar ratio between M(II) and Fe(III) cations, reaction temperature, pH value, and type/concentration of alkaline agent) [59]. The reactant undergoes precipitation (supersaturation of the metallic oxide in the solution) to reach NPs of a particular size [60]. MNCs have been studied by the coprecipitation method, which is generally synthesized from salt species such as Fe^2+^ and Fe^3+^ in an alkali solution and under non-oxidizing conditions [61].

Mehra et al. used an in situ co-precipitation method to achieve a homogeneous melamine-cyanurate (MC) distribution in a polymer matrix. As a result of this incorporation, 65% enhancement of thermal conductivity was achieved, offering a new strategy for the development of new thermally conductive materials [62].

### 3.3. In Situ Precipitation

**Figure 5:**
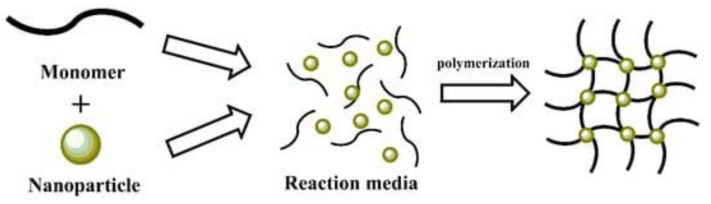
Schematic representation of in situ polymerization method. Reprinted with permission from ref. [57], MDPI, 2022.

The in situ precipitation method is widely used and reported in the literature to synthesize MNPs based on their application in many fields [49]. This method involves processes in either a solvent-free system (i.e., a bulk phase) or in a solvent-based system (including an aqueous phase) that can be purely solvent, emulsion, or suspension-based [63], which is combined with magnetic nanofillers [64], resulting in MNCs as Figure 5 shows. This simple and straightforward method loads MNPs into a polymeric matrix because this method involves the inclusion of nanoparticles into a polymer matrix in the presence of a precipitation medium [65]. The selective precipitation of small amounts of inorganic nanoparticles within the porous matrix reduces the accessible pore volume [66].

Practically, magnetic composites can be synthesized by embedding magnetic particles into a non-magnetic matrix [67]. Konwar et al. reported the synthesis of MCs using the in situ polymerization method. Firstly, the chitosan hydrogel was prepared using glutaraldehyde as a cross-linker, then the MNPs were placed to form inside the hydrogel matrix via the co-precipitation process, resulting in obtaining MNCs [68]. Wang et al. carried out fabrication via the in situ growth of nano Fe_3_O_4_ on the polydopamine (PDA)-functionalized MoS_2_ nanosheets to generate solar steam. The magnetic MoS_2_ nanosheets not only showed the long-term dispersion in an aqueous solution due to the introduction of hydrophilic PDA but also exhibited fast and effective separation from the aqueous solution with the help of the decorating nano Fe_3_O_4_, which benefit the continuously efficient solar steam generation and its good recyclability [69].

### 3.4. Blending

**Figure 6:**
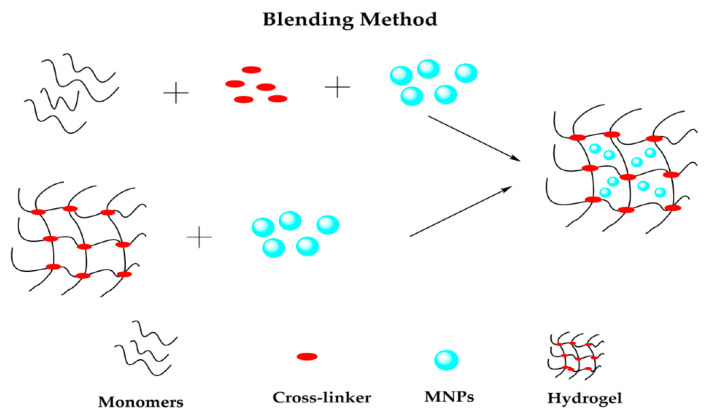
Schematic representation of blending method. Reprinted with permission from ref. [50], MDPI, 2022.

The blending method consists of the simple mixture of MNPs and the hydrogel precursor solution and the subsequent polymer cross-linking, producing hybrid magnetic loaded hydrogel networks [70]. In other words, this technique consists of the physical encapsulation of MNPs into a polymeric matrix [67] in two ways, as Figure 6 shows. Firstly, preformed MNPs are placed into the aqueous polymer solution, causing polymer chains to cross-link and encapsulate the MNPs, while the second method corresponds to MNPs and network hydrogel being made separately, and afterward, the MNPs are trapped in the network by physical interactions [50]. Such encapsulation of NPs helps stabilize NPs by preventing them from agglomerating [71]. 

Melt blending is a more adaptable technique specifically for thermoplastic NCs [72]. This technique is simple, environmentally friendly (since no solvent is needed), cost-effective, and best for mass production [73], in the presence of an inert gas such as argon, nitrogen, or neon [74]. This kind of mixing ensures that the NPs are exfoliated in the polymer matrix by fixing the MNPs within the polymer matrix just as they are in water [75,76].

Thu et al. reported preliminary results on preparing magnetic nanocomposites based on acrylonitrile butadiene styrene (ABS) and nickel nanorods (NiNRs), which were used as magnetic components. The magnetic nanocomposites were prepared by first incorporating NiNRs into the ABS matrix via a process of solution blending and then evaporation of the solvent used [77].

### 3.5. Grafting Methods

**Figure 7:**
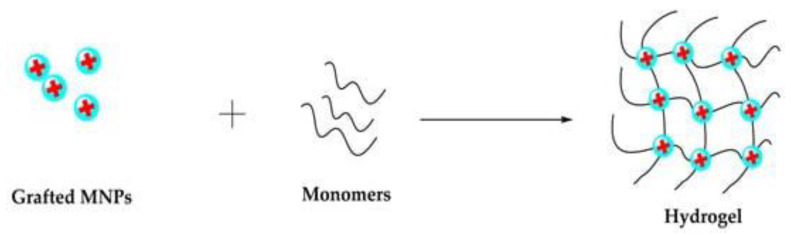
Schematic representation of grafting method. Reprinted with permission from ref. [50], MDPI, 2022.

In “grafting to” techniques, polymer chains modified with anchoring groups are used to bind to the particle surface. Likewise, the MNPs surfaces are modified with suitable functional groups that act as cross-linkers in the presence of the hydrogel precursor, allowing the formation of hydrogels with the MNPs covalently bonded to the polymeric networks [78]. Figure 7 illustrates the grafting of MNPs with monomers, forming an MNC. The magnetic polymeric matrix produced by the grafting method shows higher NP dispersion stability due to the covalent coupling [16].

In the grafting-onto method, the polymer backbone and the polymer side chains are prepared separately and coupled afterwards [79]. In this case, grafting does not involve a chain reaction [80]. Gamma radiation has been successfully developed to graft filler onto polymeric materials [81]. The grafting method is based on the surface modification of MNPs with functional groups to interact with polymer chains covalently [82].

Hu et al. designed a magnetic hydrogel made from non-toxic polyacrylamide (PAAm) hydrogel and 3-(trimethoxysilyl)propyl methacrylate-coated Fe_3_O_4_ via the grafting-onto approach. This magnetic hydrogel not only offers a relatively high modulus and toughness compared to the pure hydrogel but also responds to the magnetic field rapidly because of high magnetic particle content [50,83]. Jia et al. grafted aramid fibers, 3-aminopropyltriethoxysilane (APS), onto the epoxy matrix. The reinforced aramid fiber composite increased by 51.03%, from 36.33 to 54.87 MPa, after the modification, providing remarkable news for potential applications of aramid composites in aircraft and aerospace [84]. Islam et al. developed a novel method to graft carbon nanotubes (CNTs) onto carbon fiber (CF) via ester linkage, forming a CNT-CF hierarchical reinforcing structure. Results show that the fibrous film of CNT-CF exhibits a specific capacitance superior to pristine CF (3.5 times greater), indicating a potential application as a supercapacitor with enhanced performance [85]. In this sense, Zhao et al. scattered highly conductive carbon nanotubes into a PU matrix to produce nanocomposites for electrostatic dissipation. Dispersion of these carbon nanotubes includes the “grafting onto” method in its strategy. An addition of 1 wt.% of these components improves the tensile strength and modulus compared to pristine PU film, making it a flexible nanocomposite with low surface resistivity for aerospace coating applications [86].

All synthetic methods display advantages and disadvantages in manufacturing nanocomposites. Table 1 shows a comparison between those reviewed, covering a short description of the methods, their advantages and disadvantages, and a reference related to the topic. It is interesting to note that differences between them lie in their procedure, their obtained properties, their environmental impact, and their cost, which are key factors in obtaining nanocomposites.

## 4. Characterization of Polymer Nanocomposites for Aerospace Industry

One of the key steps of processing polymer nanocomposites and, in general, all materials, is characterization. Characterization involves two main processes: Structural and morphological analysis and properties determination. The first approach can be developed using a variety of microscopy and spectroscopy techniques, while the second depends on the individual application and includes some different techniques.

Characterizing the properties of nanostructures introduces challenges to existing analytical techniques due to some constraints related to the small size of nanostructures. This fact makes the application of well-established techniques and their manipulation difficult. In this section, several commonly used techniques are briefly detailed along with examples, starting from computational modelling (the prediction of possible properties) to the exact determination of physical and chemical properties of nanostructures by analytical techniques. It is important to highlight that, sometimes, it is compulsory to combine these analytical techniques to clarify results.

### 4.1. Computational Modelling

At the nanoscale, the interactions that can occur between the polymer matrix and the filler have a significant impact on the nanomaterial. This impact is reflected in a variety of interesting and useful properties. Computational modeling is a common way to simulate the behavior of atoms at this level, which differs significantly from what occurs at the macro scale. Molecular dynamics (MD) is one of the most common numerical methods for computational modeling because it is useful to investigate structure, dynamics, and thermodynamics of polymer nanocomposites [88,89]. MD-based methods consider interaction at the atomistic scale and apply quantum principles to predict physical and mechanical material properties by solving the equations of motion of the interaction between atoms within interatomic potentials. Generally, computational modelling is preferred over experimental techniques due to their predictive power, great accuracy, and low computational cost [90].

One of the main objectives of computational modelling is to predict properties of materials with accuracy, which is a great challenge in using conventional analytical methods. In that way, Smith et al. simulated a polymer–nanoparticle composite (PNC) model through molecular dynamics, which consisted of coarse-grained bead-necklace polymer chains and spherical nanoparticles [91]. The objective of the simulation was to determine the influence of the nanoparticle-polymer interface on the viscoelectric properties. The result was slightly surprising since, in contrast to many conventional composites already reported, the viscoelectric properties of the polymeric matrix in this material are strongly perturbed by nanoparticles and depend mainly on the interactions produced at the nanoparticle–matrix interface [91]. Furthermore, Lin et al. used molecular dynamics simulation to investigate temperature-dependent mechanical properties in graphene/PMMA polymer nanocomposites [92]. In that sense, they assumed that the graphene nanoplates were fully exfoliated in the PMMA matrix. Thus, they analyzed Young’s and shear modulus with a volume fraction variation at different temperatures. Simulation results showed that both parameters increase as temperature increases (from 300 K to 500 K), although the efficiency of graphene reinforcement decreases as its content increases [92].

Even these methods have been used to analyze the fractures within polymer nanocomposites filled with spherical nanoparticles. Hagita et al. analyzed the degree of dependence on the interactions at the nanoparticle–polymer interface through coarse-grained molecular dynamics simulations with a Poisson radius of 0.4 [93]. The experiment was conditioned by establishing attractive interactions between the polymers to observe the creation of nanovoids. Thus, it was possible to determine that the nanovoids occur in the bulk of the polymers. On the other hand, nanovoids occur at the interface surface when the interaction is repulsive. Finally, they concluded that these behaviors occur independent of the crossover densities [93].

### 4.2. Atomic Force Microscopy (AFM)

Atomic force microscopy is a technique that allows the forming of images of surfaces using sonar or a microlever. It runs through the sample doing a line-by-line scan, or in other words, it scans the sample based on position, generating an image. At distances between the detector and the sample surface (non-contact mode) greater than 10 Å, electrostatic, magnetic, capillary, and van der Waals interactions generate the topography images. On the other hand, the ionic repulsion forces are in charge of this task [94]. Therefore, this technique is advantageous to determining the distribution of nanoparticles and deformations on the polymer matrix without destroying the sample. It has been proven that the sensitivity and utility of AFM are enhanced by the addition of resonant detections. Here, we detail some resonant detectors or “tapping modes” and variations of AFM that have been useful in the characterization of polymer nanocomposites

One of the most successful variations of AFM is the addition of a measurement mode known as amplitude modulation (AM-AFM) or AC mode. In the image generation within this mode, the cantilever is dynamically excited to almost a resonant frequency. At this step, the amplitude and phase of the cantilever oscillation are measured. This measurements are used as a feedback signal to control the tip–sample distance to track sample topography [95]. However, some studies show that simultaneous excitation of higher resonance(s) of the cantilever can provide additional structural information. In this way, bimodal AFM arose as a solution operating in an “AM-FM” imaging mode, which provides a large dynamic range of measurable modulus, very fast scan speeds, and quantitative mapping of moduli and stiffness [96]. Applying this method, Nguyen et al. quantified mechanical properties at the silica/epoxy interface in this polymer nanocomposite. AM-FM images revealed the presence of an interfacial epoxy layer surrounding silica nanoparticles at a length scale of ~20 nm and an elastic modulus 2–3 times smaller than the bulk matrix. This fact confirms the stiffness of the new nanocomposite, a key factor in aerospace applications, specifically as an aircraft component [97].

Intermodulation is a phenomenon in which nonlinear systems are driven with some frequencies generated by the nonlinearity that are not present in the drive. Although intermodulation is considered an undesired effect, in some cases, intermodulation is used in an advantageous way to obtain a very sensitive, high information bandwidth mode of AFM, known as ImAFM. Ghasem Zadeh Khorasani et al. used this mode to evaluate the effect of boehmite nanoparticles on an epoxy matrix, probing the stiffness of both particles and the polymer matrix [98,99]. The high resolution of this method allowed researchers to evaluate the stiffness of epoxy matrices in samples with different contents of nanoparticles by distinguishing the force curves related to the nanoparticles and polymer. In this way, they obtained a highly resistant polymer nanocomposite, which can be useful as an aircraft component [100]. 

HarmoniX is a novel technique that uses specifically designed cantilever geometries with an out-of-axis tip. In this mode, the measurements of the topography and frictorsional deflection at different frequencies allow one to separate the two signals and achieve complete and contemporary characterization of mechanical and morphological properties in real time [101]. Using this technique, Guadagno et al. used AFM to analyze the effect of UV radiation on epoxy-based coatings properties that have incorporated Graphene Nanoplatelets [102]. For this, they used the acquisition mode “HarmoniX” to obtain nanometric-resolved maps of the mechanical properties. These maps showed that incorporating graphene nanoplatelets in low percentages in the polymeric matrix improves the mechanical stability of the samples irradiated with UV. Furthermore, the researchers point out that this beneficial effect increases with increasing nanoplatelets. Finally, they highlight the versatility of “HarmoniX” to study multiphase polymeric systems [102].

To conclude this section, Table 2 shows a comparison between all four modes presented in this section, presenting advantages and disadvantages of each one.

### 4.3. Transmission Electron Microscopy (TEM)

This sophisticated microscopy technique was born to overcome the deficiencies of light microscopes. It is a piece of equipment of vital importance to resolve the material’s structure since it allows for determining the arrangement of atoms in a sample and, in turn, determining the chemical composition. It uses a beam of very-high-energy electrons transmitted by a projector through a slide to examine the sample. Thus, the structure on the slide affects the light that passes through it, generating the desired images [94,104].

In polymer nanocomposites, filler or reinforcement is the component that confers the great majority of novel properties to the nanocomposite. Further, if filler is agglomerated, possible fracture zones could appear. For that reason, good dispersion of this filler is highly recommended if not mandatory. In this sense, TEM is a useful technique to determine how well-dispersed the filler is along the polymer matrix. For some years, this technique had been utilized for that purpose, for example, Park et al. dispersed single-wall carbon nanotube (SWCNT) bundles in a polyimide polymer matrix, obtained by in situ polymerization in the presence of sonication [105]. The resulting nanocomposites had conductive properties and were optically transparent. The analysis of the nanocomposite by TEM generated images that demonstrate that the SWNT bundle has a diameter range of 2–20 nm and shows a homogeneous dispersion on the matrix surface. The authors highlight the potential aerospace and terrestrial application of their nanocomposites due to their electrical conductivity and high optical transmission [105]. Moreover, Thakur et al. defined the dispersion of functionalized zirconium tungstate (ZrW_2_O_8_) nano-rods into an epoxy resin. In this research, the main use of TEM was to determine the functionalization of nano-rods with polydopamine, showing a thin, well-dispersed layer along the nano-rods. The final nanocomposite shows a low coefficient of thermal expansion and enhanced tensile properties, which are ideal for aerospace applications [106]. Recently, Xavier et al. incorporated 2-aminobenzothiazole (ABTA)-modified AlN nanoparticles into a polyurethane matrix. TEM was used to determine the degree of dispersion of filler. TEM images showed that only AlN nanoparticles in the matrix produce agglomeration, whereas including ABTA/AlN nanoparticles provides a good distribution over the matrix due to the functionalization of AlN nanoparticles by ABTA. This inclusion provides new properties such as hydrophobicity and corrosion resistance against chloride, which make this nanocomposite a potential candidate to be incorporated as coating in aircraft [107]. If we compare these three projects, with a difference of 20 years between them, the need to achieve mechanically resistant materials with improved properties is evident.

Other important factors to be determined by TEM are the orientation, size, and crystallinity of the filler. These characteristics could influence the performance of the nanocomposite, conferring it enhanced or worsened mechanical properties. In the case of the orientation, this could influence the response in the presence of an external stimulus such as magnetism. Precisely, Yoonessi et al. synthesized oriented hybrid nickel tethered graphene polyimide resin nanocomposites with different degrees of orientation, using the in situ magnetic field solvent casting method [108]. TEM was used to determine the alignment of the polymer matrix’s nickel-tethered graphene nanosheets and stacks. According to the images generated by the technique, when the superparamagnetic nickel nanoparticles are properly aligned in the direction of the applied magnetic field, they also align the graphene stacks to which they are tethered [108]. To determine the size of filler content, Zuo et al. analyzed TEM micrographs, observing that Li_0.35_Zn_0.3_Fe_2.35_O_4_ particles embedded into polyaniline have a size of 500 nm–1.6 μm and a lattice spacing of 0.25 nm. This latest result is in accordance with XRD results. This nanocomposite shows enhanced microwave absorption [109]. These properties could be aptly used in high-performance applications such as the aerospace industry, due to the high mechanical resistance achieved by these novel nanocomposites. Table 3 shows information obtained by TEM in reviewed articles.

### 4.4. Raman Spectroscopy

Within nanotechnology, one of the challenges is to develop, control, and design nanomaterials according to certain needs. These needs, in turn, are related to nanoscale properties. All this is achieved with an amalgamation of parameters that must be taken into account to synthesize the nanomaterial (reagents, conditions, methodology, etc.). Once the structure is obtained, there is a need to characterize it. However, not all techniques can provide reliable structural information, such as Raman Spectroscopy (RS) [110]. RS is a technique that uses the interaction of light with matter to obtain information about the composition or characteristics of a material. RS is a unique tool that allows for mapping of the nanophases dispersed in a polymeric matrix, employing the intramolecular and intermolecular vibrations of the dispersion [94,110]. The information obtained by this technique is complemented by that provided by TEM.

This technique is extremely useful to verify the inclusion of inorganic fillers (but not limited to them) since molecular vibrations between organic (matrix) and inorganic (filler) atoms occur at low wavenumbers that commonly appear in the infrared region. Based on this premise, Pérez et al. determined the orientation distribution function (ODF) in a uniaxial-axially symmetric system using polarized RS [111]. With the development of this method, the main objective was to quantify the alignment of single-wall nanotubes in a polymeric matrix. In addition, certain nanocomposites obtained by different methods were analyzed by RS and other dielectric spectroscopies. It was possible to obtain information that allows the optimization of the parameters to achieve unique electrical and mechanical properties in SWNT nanocomposites [111].

Furthermore, preliminary synthesis of thermoset nanocomposites with aligned carbon nanotubes was developed by Haibat et al. [112]. The fabrication was carried out using magnetic fields, in an energetically efficient and fast manner. These nanotubes were Ni-coated, and RS quantified their dispersion. This technique made it was possible to verify that the obtained nanocomposites have excellent transport and magnetic properties [112].

### 4.5. Thermal Characterization

In aerospace applications, the thermal behavior of its components is a crucial parameter to be considered, due to extreme conditions of heat and the performance required [113,114]. Thermogravimetric analysis (TGA) and Differential Scanning Calorimetry (DSC) are the most common techniques used to determine the thermal behavior of polymers. Both are complementary analyses, giving us information such as thermal degradation at the determined temperature and thermal transitions (T_g_, melting point, etc.). Some studies were performed with both techniques, which has allowed for defining whether a polymer nanocomposite is suitable for aerospace applications.

The DSC technique is usually applied in the determination of α-transitions in polymers and their corresponding composites. These transitions are strongly related to the Brownian motion that occurs during the transition from a vitreous to rubbery state, and the relaxation of the dipoles related to it [115]. 

In that sense, Zhang et al. synthesized iron nanoparticle-reinforced polyacrylonitrile (PAN) nanocomposites using a simple and eco-friendly solvent extraction method [116]. DSC and TGA can verify the strong interaction between the filler (iron nanoparticles) and the polymer matrix (polyacrylonitrile). This conclusion is reached because when the samples are heated up to 300 °C, there is a higher weight loss of the polymer nanocomposites compared to the pure polymer matrix. The thermal stability of polymer nanocomposites is accomplished by the dispersion of iron oxide nanoparticles, resulting in an enhancement in the thermal performance, which could be beneficial for high-performance conditions [116].

Guo et al. developed solid magnetic epoxy polymer nanocomposites using polypyrrole as a coupling agent coating on magnetite (f-Fe_3_O_4_) nanoparticles [117]. New nanocomposites show good novel properties such as improved magnetic properties, enhanced mechanical tensile strength, and an increased glass transition temperature. This last property was evaluated by DSC, observing a progressive increase in T_g_ from 87.4 °C (cured epoxy PNCs with an f-Fe_3_O_4_ loading of 5 wt.%) to ~150 °C (cured epoxy PNCs with an f-Fe_3_O_4_ loading of 30 wt.%), due to the cross-linking between the inorganic nanoparticles and epoxy matrix [117].

Zotti et al. synthesized metal core/polymeric nanoparticles from an acrylamide complex of iron and nickel nitrates using controlled thermolysis [118]. The nanoparticles were used as a filler for epoxy resin nanocomposites. Results from TGA analysis proved the effect of iron particles is relevant in the thermal stability of polymer nanocomposites, observing a lower second degradation temperature in comparison with those of the corresponding neat resin [118].

Govindaraj et al. developed new polymer-carbon nanotube nanocomposites from 2,4-bis(4-aminophenylamido)-6-chloroquinazoline, pyromellitic dianhydride, and functionalized carbon nanotubes by a microwave method [119]. Using DSC, they can verify the improvement in the Tg values in modified polyimide. For example, pristine polyimide has a T_g_ of 293 °C, polyimide modified with carbon nanotubes has T_g_ = 338 °C, polyimide modified with acid and carbon nanotubes has T_g_ = 349 °C, and polyimide/amine-carbon nanotubes nanocomposites has T_g_ = 375 °C. These achieved values confirm the presence of hydrogen bonds and covalent bonds in the nanocomposites. This fact indicates that the incorporation of the filler resulted in an increase in the glass transition temperature due to the restriction in the movement of the polymer chains and the formation of a network structure acting as a support for the polymer chains throughout the composites. These are promising results for the aerospace and electronics industries, where high Tg, dielectric constant, and flame retardancy are required [119].

Finally, Aktitiz et al. produced nanocomposite structures of resin, via the additive manufacturing method, doped with Fe_2_O_3_ nanoparticles [120]. Fe_2_O_3_ nanoparticles were added to the matrix in different concentrations. Results revealed that the inclusion of Fe_2_O_3_ in the polymer matrix improves the thermal stability of the whole material, especially in the 1% reinforcement. The authors explain that this fact could be explained by the capacity of nanoparticles to act as a catalyzer of the curing reaction and play the role of thermal stabilizer. This improvement can reorganize the polymer chains’ degradation, movement, and thermal cracking. This work concluded that 3D-printed nanocomposites doped with Fe_2_O_3_ nanoparticles could be considered a suitable material for applications with good thermal and magnetic properties desired, such as aerospace applications [120].

### 4.6. X-ray Diffraction

X-ray diffraction (XRD) is a powerful technique for material characterization, especially for crystallographic materials including polymers, composites, and polymer matrix nanocomposites. Using this technique, it is possible to determine the crystalline and amorphous phases of polymers and its fillers. Some of its main advantages include its simplicity, reliability, the interesting quantitative information that it provides, and its nondestructive nature. For example, for the semiconductor industry, XRD has been used to determine crystallite size, lattice strain, structural composition, and crystal orientation. The diffractogram obtained by XRD is similar to a fingerprint of the crystal arrangement of a material [121].

Another advantage of XRD information is that it comes with minimal sample preparation and relatively easy interpretation of the results. However, these results can be complemented with other information of other techniques such as TEM. In this context, some experiments have been performed to verify the crystallinity and crystallographic structure of reinforcement. For example, Sanida et al. used XRD to verify the crystallographic structure of ZnFe_2_O_4_ nanopowder incorporated into epoxy resin. They found five major peaks (220, 311, 400, 511, and 440), which are consistent in both position and intensity with a cubic, spinel cryallographic structure with a Fd3m space group and lattice parameter a = 8.4411 Å [122]. One year later, this research group probed the correct incorporation of Fe_3_O_4_ nanoparticles in the epoxy matrix, forming a polymer-magnetic nanocomposite. For this, they performed X-ray diffraction on the nanocomposite, obtaining peaks ((220), (311), (400), (422), and (440)) and their corresponding intensities consistent with the inverse cubic spinel crystal structure of magnetite, the Fd3m space group, and lattice parameter a = 8.3582 Å. The average crystal size was 44 ± 3 nm. Moreover, they verified that the intensity of the diffraction peaks increases according to the filler content in the nanocomposite [123]. If we compare both studies, it is interesting to note that the incorporation of Zn in the first case does not alter the crystallographic structure of filler, but both fillers contribute to providing improved mechanical properties to the final polymer nanocomposite. 

Further information provided by XRD is related to the homogenous dispersion of filler into the polymer, reflected in the degree of crystallinity of the filler and matrix. Sometimes, the crystallinity of a matrix could be partially or completely affected by the presence of reinforcement that has another crystal structure. A homogenous distribution of the filler will guarantee a homogeneous crystallinity along the matrix. Furthermore, XRD can provide information on the purity of a dispersed filler, observing whether only one crystal structure is present in XRD patterns. For this reason, revisiting an example from the TEM section, Zuo et al. took advatage of all of these possibilities and corroborated that a high-purity LZFO nanoparticles was dispersed into the PANI matrix. They achieved this conclusion from the observation of the diffraction pattern, which showed characteristics peaks assigned to the reflection of (220), (311), (400), (422), (511), and (440) planes. This pattern confirmed that LZFO particles with a spinel structure and space group Fd3m were well-dispersed in the polymer matrix surface [109]. Mohamed et al. corroborated, via XRD, the correct dispersion of annealed ZnS nanoparticles into a PVA matrix. They achieve this conclusion since the XRD pattern indicates that there is no difference between pure PVA and loaded ZnS along the entire nanocomposite, showing an expected partial crystallinity structure (2θ = 21°) [124]. Using the diffraction pattern provided by XRD, Guo et al. verified the inclusion of Fe_3_O_4_ and CoFe_2_O_4_ magnetic nanoparticles in a PANI matrix. This inclusion was seen in nanocomposites with different weight percentages of the inorganic compound into the matrix and provided an enhanced dielectric property that could be useful in EMI shielding [125]. Recently, Mustafa et al. improved the mechanical properties of an epoxy resin, including ZrO_2_ and Y_2_O_3_ nanoparticles. Those nanocomposites improved with ZrO_2_ show a diffraction pattern corresponding to crystal planes of the monoclinic zirconia phase, and those obtained via the inclusion of Y_2_O_3_ are related to the cubic crystal structure. Another interesting conclusion is that the reduction in the intensity of characteristic peak of epoxy resin upon adding NPs indicates an improvement of mechanical properties, giving it more stiffness and rigidity [126].

All of these studies exemplify the versatility of the X-ray diffraction technique, which range from verifying the inclusion of fillers to the structural determination of crystallinity and dispersion of reinforcement into the matrix. However, it is necessary to verify information provided by XRD using more techniques detailed in this section. Table 4 summarizes examples reviewed in this section, with the main information obtained from XRD patterns.

Table 5 summarizes characterization techniques of polymer matrix nanocomposites, including general information that these techniques provide and useful references related to them.

## 5. Aerospace Applications

The development of nanocomposites in some fields, such as military and aerospace, is doubtless. The use of these advanced materials as part of aerospace structures are essential to obtain effective planning, development, accreditation, operation, and maintenance of aircraft structures [127]. Some phenomena such as electromagnetic interference, corrosion, and icing can induce structural damage and could compromise one or all of the aforementioned steps. For this reason, in this section, three relevant applications of polymer nanocomposites are detailed to avoid these issues, namely, EMI shielding, coatings and paints, and SHM. 

### 5.1. EMI Shielding

Polymeric matrix composites coated with metallic magnetic nanoparticles have different applications in the aerospace industry; one of the most important is electromagnetic interference (EMI) shielding materials. Currently, around the world, there are many devices, such as transmitters, for navigation, communication, or satellite propagation that generate an electromagnetic interference environment that may affect the electronic devices that an aircraft requires to navigate [128]. Therefore, metal materials appeared as an option to shield the aircraft from electromagnetic radiation due to their high electrical conductivity [129] and their generation of an effect similar to the Faraday cage [128]. However, its materials have a big problem because they have high weight values and corrode easily [130]. To overcome these problems, the polymer matrix coated with metallic magnetic nanoparticles combine the dielectric and magnetic behavior of metals and carbon material to generate a very efficient, corrosion-resistant, and lightweight EMI shield [131].

A poly(vinylidene fluoride) (PVDF)/Fe_3_O_4_/carbon composite film was synthesized to generate electromagnetic interference (EMI) shielding material. Cheng et al. developed an EMI shielding system based on radiation absorption and converted it into thermal energy. It is a hybrid system composed of two phases that provide different characteristics: Carbon material (dielectric loss) and ferromagnetic nanoparticles (magnetic loss). The synergy of both phases allowed them to obtain a value of shielding effectiveness (SSE) of 35.6 dB [131].

Tang et al. constructed a corrosion-resistant, lightweight, and electromagnetic interference (EMI) shielding polymeric composite. This composite consisted of poly (lactic acid) particles coated with Ag nanoparticles and deposited over a poly(caprolactone) multi-walled carbon nanotubes surface. In that system, the metallic interface enhanced the multiple scattering, allowing an SSE value of 43.4 dB [130].

Jalali et al. researched the effect of electromagnetic shielding by including metal (iron, cobalt, nickel, and iron oxide) nanoparticles into the polymeric matrix (epoxy resin and carbon mesh) to generate high-strength and non-heavy EMI shielding material for aircraft applications. The results showed that iron nanoparticles proved to contribute to a high SSE value equal to 45 dB [128]. Velhal et al. achieved 89% microwave absorption and an SSE value of 37.49 dB employing a polypyrrole/Ba_0.6_Sr_0.4_Fe_12_O_19_ composite. Therefore, this material appears low cost and resistant as EMI shielding material [132].

Zhang et al. developed electromagnetic interference (EMI) shielding polymeric membrane. This material was synthesized by employing the fiber of polyacrylonitrile (PAN) and polyurethane (PU) generated by electrospinning and coated with Ni-Co nanoparticles. Thanks to its intrinsic conductivity and magnetism, this alloy provided an EMI shielding effect to the membrane. The characterization study showed that the shielding effectiveness (SSE) and shielding effectiveness divided by the thickness of material (SSEt) were 77.8 dB and 7325.8 dB cm^2^ g^−1^, which is superior to other reports. This material mainly protects electrical devices from radiation pollution and has potential applications in the aerospace industry [129]. Besides, a highly efficient EMI film with an SSE value equal to 93.8 dB was synthesized by Xu et.al. They used cellulose nanofibril functionalized with polydopamine as a composite matrix. Then, silver nanoparticles were incorporated into the film by chemical methods [133].

Gu et al. developed a set of polylactide-based polyurethane (Fe_3_O_4_/PLAUs) shape-memory nanocomposites that exhibit a speedy magnetic response [134]. The homogeneous distribution of Fe_3_O_4_ nanoparticles was achieved using oleic acid, which is effective for nanocomposites with low nanoparticle content. The recovery of the original shape of the nanocomposites after being exposed to a magnetic field occurs in short times between 11 and 15 s. In an alternating magnetic field, it recovers in times between 24 and 60 s. They also note that the rapid shape recovery in an alternating magnetic field indicates a great potential for biomedical applications and the aerospace industry since the material is designed for high-performance uses [134].

On the other hand, Vertuccio et al. designed smart coatings of epoxy-based carbon nanotubes to apply them to industrial Carbon-Fiber-Reinforced Plastics (CFRPs) [135]. These materials are commonly used in the aeronautical industry and require high-performance parameters. One of the most important conclusions reached was that the gage factor of the CFRP coated with the new coating is 4.7, which is the highest value obtained for thermosetting resins. This high value makes it ideal for applications where a tremendous mechanical performance of the primary structures of the aerospace industry is required [135].

Liang et al. prepared a nanocomposite for EMI shielding from three-dimensional Fe_3_O_4_ decorated carbon nanotubes/reduced graphene oxide foam and epoxy as a polymer matrix. The EDFe_3_O_4_-CNTs/rGF/EP nanocomposite exhibited an extraordinary EMISSE value of 36 dB within the X-band range, which converts it into a potential candidate for EMI shielding applications [136].

### 5.2. Coatings and Paints

Corrosion protection is a key aspect in the aerospace industry. This is because corrosion impacts safety, aircraft availability, and the structural integrity of aircraft structures. For this reason, the design of active protective coatings plays an important role in the corrosion protection scheme. Although corrosion is a very important parameter, it is difficult to take into account because the prediction of corrosion initiation and the extent of possible structural damage is hard to determine [137]. However, there is an approach that has gained some attention. This is the development of coatings and paints based on polymer matrix nanocomposites reinforced with an inorganic phase. Some advantages of this approach have been reported, which include low cost, relatively easy fabrication, and the obtention of resistant and lightweight materials [138]. In this sense, Kang et al. prepared CoFe_2_O_4_/PANI magnetic additives, using oleic acid as a dispersing agent, to be used as a potential corrosion protective agent. This nanocomposite was included in an epoxy coating. After 100 days of immersion in acidic or saline conditions, the electrochemical impedance spectroscopy showed promising values (9.47 × 10^10^ and 4.81 × 10^10^ Ω, respectively). These results show that the inclusion of the nanocomposite significantly improves the coating resistance and anticorrosive properties [139].

Asif et al. developed a magnetic nanocomposite based on hydroxyapatite nanoparticles (MHAPs) and polyurethane (PU) with anticorrosive properties. This behavior was examined using immersion in a NaCl solution and electrochemical methods. Contact angle studies show a hydrophobic nature (105°), which increases with MHAP content, obtaining a maximum value at 3% of filler [140].

Fazli-Shokouhi et al. reported paint coatings from epoxy-based polyaniline (PANI)-graphene oxide nanosheets (GONs), obtained by the in situ polymerization process. PANI-based nanocomposites with different fractions of GONs confer anticorrosion and antifouling properties to the epoxy resin. The epoxy-12 wt.% PANI-GON coating shows the highest corrosion resistance of 2.70 × 10^6^ Ω cm^2^ after 192 h of immersion in saline water. This paint coating inhibits the diffusion process against a corrosive environment [141].

Zhang et al. explored the anti-icing properties of nanocomposites based on amino-modified magnetic Fe_3_O_4_ nanoparticles (MNP@NH_2_) and an amphiphilic P(poly(ethylene glycol) methyl ether methacrylate-*co*-glycidyl methacrylate) copolymer, followed by infusion with some polyols. This interesting material exhibit an antifrosting property reflected in the capacity of postponing frost formation for as long as 2700 s at –18 °C. Furthermore, the crystallization point of water can be reduced to −36.8 °C and has an extremely low ice adhesion strength (0.1 kPa), which makes it a good candidate for anti-icing and deicing aerospace requirements [142]. In this way, Hou et al. developed a PVDF-HFP/SiO_2_/CNTs nanocomposite coating with anti-icing and superhydrophobic properties. This material shows exceptional water repellency reflected in a contact angle of up to 168° and a slide angle lower than 2°. Moreover, the anti-icing performance was demonstrated by the ice formation process on aluminum film being delayed by more than 300 s at −19°. These properties can be useful in the aerospace industry with its extreme polar climate requirements [143].

### 5.3. Structural Health Monitoring

Structural Health Monitoring (SHM) is an important method used to determine the integrity of structures involved in aerospace applications. This method includes multidisciplinary areas such as sensors, materials, signal processing, and interpretation [144,145]. It is important to highlight that SHM is not only focused on detecting structural failures in the components but also provides a prompt signal of this damage. These indications will allow one to take remedial strategies to avoid these problems [145]. Piezoelectric materials based on inorganic/organic composites are used as suitable components for aerospace and aeronautical monitoring sensors. D’Ambrogio et al. developed a piezoelectric composite made of lead zirconate titanate (PZT) as a filler, embedded in a poly(dimethyl siloxane) (PDMS) matrix. This new material has a superior piezoelectric behavior, even at high temperatures (200 °C), for a configuration of 1–3 of PZT/PDMS. For this reason, these researchers concluded that this composite has great potential to be part of a sensor in aircraft ball bearings for condition monitoring [146].

To finish this section, Table 6 shows a summary of detailed aerospace applications of polymer nanocomposites with their achieved properties and references about them.

## 6. Future Research Trends

The use of polymer matrix nanocomposites as aircraft components in the aerospace industry is, undoubtedly, growing. Now, all efforts are focused on the replacement of metal components with polymeric components, which include polymer matrix composites. Additionally, nanotechnology makes its contribution by transforming composites into nanoscale composites, i.e., nanocomposites, with improved properties that cannot be achieved at the macroscale. Despite these enormous advances and efforts, it is necessary to focus on the challenges and convert them into opportunities and future research trends in the development of polymer nanocomposites.

As is well described in this review, the addition of filler into the polymer matrix confers the great majority of properties that nanocomposites show or improves the inherent properties of matrix. For this reason, a good dispersion of nanoparticles into the polymer matrix is necessary to obtain adequate results and avoid local stress concentrations, i.e., agglomeration, which eventually result in product failure. Homogeneous dispersion is vital for the optimum performance of a nanocomposite. Achieving this distribution will depend on the correct combination of the synthetic method to obtain the nanocomposite, as well as the nature of the filler. Furthermore, enhancement of the polymer–reinforcement interaction via the modification of the morphology, optimum aspect ratio, surface roughness, or by introducing functional groups will allow for improved mechanical properties. It will provide the innovative approaches, ideas, and processes necessary to overcome this challenge. 

Another challenge is related to the gap that has always existed between industry and academy. As this review has shown, the great majority of synthesized polymer nanocomposites have been obtained at the laboratory level, exhibiting all the potential aerospace applications. However, it is necessary to create industrial processes to make them more commercially available. Manufacturing processes of nanocomposites are economical for low volume production, that is, at a laboratory scale, but higher prices are expected at the industrial level. The cost of feedstock production, post-processing, and energy consumption associated with these new processes will need to be taken into account. It is necessary for the creation of these industrial synthetic methods to obtain nanocomposites to be proven in more realistic scenarios, as part of aircraft components, with all the variables that can affect the expected properties.

Finally, but no less important, the development of new polymer nanocomposites and their industrial processes must be in accordance with environmental aspects. In fact, humanity is aware of the necessity to reduce the environmental impact that some laboratory and industrial manufacturing processes have, including the manufacturing of polymer nanocomposites. This fact has led governments to establish more severe regulations and the scientific community to explore new environmentally friendly polymer nanocomposites. The first goal to achieve is to find eco-friendly solvents to synthesize those materials. The impact that solvents have had on environmental pollution is considerable, and it is compulsory to reduce this environmental footprint. In this way, polymer nanocomposites obtained through the use of eco-friendly materials and techniques, as well as the inclusion of filler in biopolymers, have emerged as very promising materials due to the excellent properties obtained, which include ease of processing, as well as impressive physico-chemical properties [147,148]. This provides a new opportunity for the development of these materials, and it will be necessary to study and show that eco-friendly polymer nanocomposites can be applied in the aerospace industry.

## 7. Conclusions

Progressively, polymeric nanocomposites have been contributing significantly to various industries where certain requirements must be met. In particular, in the aerospace industry, where the use of metal-based components still prevails, the advancement of polymer nanocomposite materials has resulted in obtaining lighter components that are also resistant to various phenomena such as fatigue, corrosion, and radiation. Some synthetic and manufacturing processes have been used to develop nanocomposite parts, while newer techniques are being analyzed and tested consistently. Each technique has its advantages and limitations, considering the complexity of the steps to be followed and that the choice of technique will influence the quality of the final nanocomposite. One of the most important factors to take into account is the homogeneous distribution of the reinforcement throughout the entire polymeric matrix since this will guarantee the correct deployment of the aforementioned characteristics. To verify these aspects, the proper use of the different characterization techniques detailed in this review will be necessary, such as computational modeling, AFM, TEM, RS, DSC, TGA, and XRD, which, together, will provide a wide range of information for the elucidation of the material. Although these techniques have helped determine that the inclusion of metal oxide nanoparticles helps to considerably improve the performance of the nanocomposite, carbon nanotubes have also demonstrated their ability to display superior mechanical properties and provide stability of strength and reduced fatigue stress. This implementation could certainly revolutionize the aerospace industry.

Polymer nanocomposites have been tested successfully as part of aircraft components, with applications of high performance demanding the employment of structural materials, which nanocomposites seem to fulfill sufficiently. The implementation of those nanocomposites has considerably improved the structural capabilities of specific components, meeting the stringent material and manufacturing requirements of the aerospace industry. For EMI shielding applications, a great variety of polymer nanocomposites have been synthesized, with the most promising being the Ag/Cellulose-polydopamine nanocomposite, which achieves 93.8 dB with improved mechanical properties. Furthermore, for corrosion-resistant coatings, the SiO_2_-CNTs/PVDF-HFP nanocomposite is remarkable, which is a robust superhydrphobic coating (>160°) with anti-icing properties that delayed this phenomenon for more than 300 s at −19 °C. However, in SHM applications, an important process in the aerospace industry, there is still much left to do, highlighting the the PZT/PDMS nanocomposite with its superior piezoelectric behavior maintained at higher temperatures and a lower poling electric field. These prominent examples evidence the potential use of polymer nanocomposites as a part of aircraft components and the impact that these materials could have in the near future.

The future of polymer nanocomposites in the aerospace industry looks quite promising. This is due to aerospace technology and its ever-increasing challenges requiring materials with long-lasting properties, making it easy to choose them over conventional metals or their alloys. However, synthetic processes must be created on both laboratory and industrial scales that allow the full potential of these materials to be exploited, thus ensuring a homogeneous distribution of the filler. Finally, these processes must be created based on eco-environmental parameters in order to reduce the increasingly evident environmental damage to our planet. If all of these factors are taken into account, there is no doubt that polymer nanocomposites will soon completely replace metal-based aircraft components.

## Figures and Tables

**Table 1:** Summary of synthetic methods for polymer nanocomposites with their advantages and disadvantages.

Synthetic Method	Brief Description	Advantages	Disadvantages	References
Molding	A polymeric stamp is placed in contact with a precursor of a solid material	Absolute sizes and aspect ratios can be replicated Ability to mold patterns	The resolution of molding is limited by the inherent atomic and molecular graininess of matter	[52]
Co-precipitation	Reducing a mixture of metallic ions using a basic solution at low temperature and in an inert atmosphere	High yield and high product purity No organic solvent is needed Easy reproducibility Low cost	Properties of obtained particles (size, shape, and composition) are highly dependent on reaction parameters Need to add low molecular weight surfactants to stabilize obtained nanoparticles	[58]
In situ precipitation	Nanoparticles dispersed in a monomer or monomer solution and polymerization under standard techniques	Better exfoliation achieved in comparison with other methods	Need of adequate solvent, including water and mixtures. Not eco-friendly	
Blending	Polymer melted with a desired amount of filler in presence of an inert gas and heat	Easy Eco-friendly Compatible with industrial processes (ideal for mass production and cost-effective)	There are chances of polymer matrix degradation	[64]
Grafting	Dispersion of nanoparticles along the surface polymer matrix initiated by radical polymerization	Covalently grafted filler on the solid surface Good control over the polymer molecular weight Application in a wide range of monomers, obtaining nanocomposites with different functionalities Good dispersion of filler	Harsh industrial operation conditions Few available polymerization methods	[87]

**Table 2:** AFM modes for characterization of polymer nanocomposites with their advantages and disadvantages.

AFM Modes	Advantages	Disadvantages	Ref
AM AFM	Minimal sample damage. Low lateral forces. High-resolution. Very fast imaging capabilities.	Difficulty in quantifying sample mechanical properties	[97]
AM-FM AFM (Bimodal)	Ability to vary and optimize the parameters without affecting topographical acquisition. Higher uniform sensitivity.	Higher cost	[103]
ImAFM	Quantitative force measurements with nanoscale resolution.	Higher cost	[99]
HarmoniX AFM	Delivering precise property maps in real time and with high resolution. Effective in the characterization of soft materials, thin films, small particles or domain within a bulk solid	Higher cost	[101]

**Table 3:** Information provided by TEM for polymer nanocomposites.

Matrix	Filler	Properties	Information Provided by TEM	Ref
Polyimide	SWCNT	Conductive and electrical properties	Degree of dispersion and size diameter (2–20 nm)	[105]
Epoxy resin	ZrW_2_O_8_ nano-rods	Low coefficient thermal expansion and enhanced tensile properties	Degree of dispersion of filler	[106]
Polyurethane	ABTA/AlN nanoparticles	Hydrophobicity and corrosion resistance against chloride	Degree of dispersion	[107]
Polyimide	Ni tethered graphene	Magnetic responsive nanocomposites	Degree of alignment	[108]
Polyaniline	Li_0.35_Zn_0.3_Fe_2.35_O_4_ nanoparticles	Enhanced microwave absorption	Degree of crystallinity, size and lattice spacing	[109]

**Table 4:** Information provided by XRD for polymer nanocomposites.

Matrix	Filler	Information Provided by XRD	Ref
Epoxy resin	ZnFe_2_O_4_ nanopowder	Crystallographic data	[122]
Epoxy resin	Fe_3_O_4_ nanoparticles	Crystallographic data	[123]
PANI	Li_0.35_Zn_0.3_Fe_2.35_O_4_ nanoparticles	Crystallographic data Crystallinity and purity of filler Homogeneous dispersion	[109]
PVA	ZnS nanoparticles	Crystallographic data Homogenous dispersion Crystallinity of filler	[124]
PANI	Fe_3_O_4_ and CoFe_2_O_4_ magnetic nanoparticles	Crystallographic data Homogenous dispersion	[125]
Epoxy resin	ZrO_2_ and Y_2_O_3_ nanoparticles	Crystallographic data Homogenous dispersion Structural information	[126]

**Table 5:** Summary of characterization techniques for polymer nanocomposites.

Characterization Technique	Information Provided by the Technique	Ref
Computational modelling	Prediction of potential properties of nanocomposite	[93]
AFM	Images of surface morphology of nanocomposite	[94]
TEM	Structural arrangement of nanocomposite	[94,104,105]
Raman Spectroscopy	Structural composition of nanocomposite about covalent binding between organic and inorganic components	[112]
DSC and TGA	Thermal behavior of nanocomposite	[84,85,115]
X-ray Diffraction	Composition and degree of crystallinity of nanocomposite	[121,123,124,125]

**Table 6:** Summary of aerospace application of polymer nanocomposites.

Application of Nanocomposite	Polymer Matrix	Reinforcement	Properties	Ref
EMI shielding	PVDF	Fe_3_O_4_/carbon	Lightweight	[131]
	PLA	Ag	Multiple scattering	[130]
	Epoxy resin	Iron, cobalt, nickel, and iron oxide	High strength and non-heavy	[128]
	PPy	Ba_0.6_Sr_0.4_Fe_12_O_19_	Low-cost and resistant	[132]
	PAN and PU	Ni-Co	Intrinsic conductivity and magnetism	[129]
	PLAUs	Fe_3_O_4_	Shape recovery in a magnetic field	[134]
	Epoxy resin	CNTs	High resistance	[135]
	Epoxy resin	EDFe_3_O_4_-CNTs/rGF	High EMISE value	[136]
Coatings and paints	PANI	CoFe_2_O_4_	Anticorrosive properties	[139]
	PU	MHAPs	Anticorrosive properties	[140]
	Epoxy-PANI	GONs	Anticorrosion and antifouling properties	[141]
	P(poly(ethylene glycol) methyl ether methacrylate-*co*-glycidyl methacrylate)	Fe_3_O_4_	Antifrosting property	[142]
	PVDF-HFP	SiO_2_/CNTs	Anti-icing and superhydrophobic properties	[143]
SHM	PMDS	PZT	Superior piezoelectric behavior	[146]

## Data Availability

Not applicable.
